# A qualitative study of minority ethnic women’s experiences of access to and engagement with perinatal mental health care

**DOI:** 10.1186/s12884-022-04698-9

**Published:** 2022-05-18

**Authors:** Sabrina Pilav, Kaat De Backer, Abigail Easter, Sergio A. Silverio, Sushma Sundaresh, Sara Roberts, Louise M. Howard

**Affiliations:** 1 Oxleas NHS Foundation Trust, Bexley, Bromley and Greenwich Perinatal Mental Health Service, Queen Mary’s Hospital, I Block, Frognal Avenue, Sidcup, DA14 6LT, London, UK; 2 Department of Women & Children’s Health, School of Life Course Sciences, Faculty of Life Sciences & Medicine, King’s College London, 10th Floor North Wing, St., Thomas’ Hospital, Westminster Bridge Road, London, SE1 7EH UK; 3 Section of Women’s Mental Health, Institute of Psychiatry, Psychology and Neuroscience, King’s College London, 16 De Crespigny Park, London, SE5 8AF UK

**Keywords:** Perinatal mental health, Minority ethnic women, Maternity services, Qualitative analysis

## Abstract

**Background:**

Approximately one in five women will experience mental health difficulties in the perinatal period. However, for a large group of women, symptoms of adverse perinatal mental health remain undetected and untreated. This is even more so for women of ethnic minority background, who face a variety of barriers which prevents them from accessing appropriate perinatal mental health care.

**Aims:**

To explore minority ethnic women’s experiences of access to and engagement with perinatal mental health care.

**Methods:**

Semi-structured interviews were conducted with 18 women who had been diagnosed with perinatal mental health difficulties and who were supported in the community by a specialist perinatal mental health service in South London, United Kingdom. Women who self-identified as being from a minority ethnic group were purposefully selected. Data were transcribed verbatim, uploaded into NVivo for management and analysis, which was conducted using reflective thematic analysis.

**Results:**

Three distinct overarching themes were identified, each with two or three subthemes: ‘Expectations and Experiences of Womanhood as an Ethnic Minority’ (Shame and Guilt in Motherhood; Women as Caregivers; Perceived to Be Strong and Often Dismissed), ‘Family and Community Influences’ (Blind Faith in the Medical Profession; Family and Community Beliefs about Mental Health and Care; Intergenerational Trauma and Family Dynamics) and ‘Cultural Understanding, Empowerment, and Validation’ (The Importance of Understanding Cultural Differences; The Power of Validation, Reassurance, and Support).

**Conclusion:**

Women of ethnic minority background identified barriers to accessing and engaging with perinatal mental health support on an individual, familial, community and societal level. Perinatal mental health services should be aware ethnic minority women might present with mental health difficulties in different ways and embrace principles of cultural humility and co-production to fully meet these women’s perinatal mental health needs.

**Supplementary Information:**

The online version contains supplementary material available at 10.1186/s12884-022-04698-9.

## Background

The perinatal period (through pregnancy and up to one year after birth) is a time of adjustment for all mothers. However, an estimated 10–20% of women will experience adverse mental health in the perinatal period [1, 2]. Unfortunately, a large proportion of women experiencing symptoms are not identified, particularly those from minority ethnic backgrounds [3], with evidence suggesting these women have an increased risk of perinatal mental health difficulties [4–7] and are more likely to report adverse childhood experiences (ACEs) [8]. In addition, minority ethnic women who report high rates of daily experiences of racism and have been found to have increased risk of perinatal depression, adverse obstetric outcomes such as preterm birth [9] and overall mortality in the perinatal period [10]. This has raised further concerns that Black, Asian, and minority ethnic women may be more likely to fall through gaps in healthcare services [11].

There is a variety of evidence to suggest access to and experiences of healthcare services, including maternity and perinatal mental health services, are far from equitable [12], but limited research has aimed to understand the barriers which prevent women from different minority ethnic backgrounds accessing care [13–16], or to explore their experiences in the wider context of their lives [17].

The few studies which have investigated barriers to accessing services have identified both system level barriers in primary care [13], psychological therapy services [18–20], and maternity services [21, 22]; as well as psycho-social, cultural, and interpersonal barriers [22, 23] and include but are not limited to negative attitudes towards mental illness, inadequate resourced services, language barriers, differences in cultural values and fragmentation of services [13, 21–23]. In England, women from Black African, Asian, and White Other ethnic backgrounds access community perinatal mental health services significantly less than White British women, and also report higher percentages of women detained under the Mental Health Act [24]. Research addressing experiences of postnatal depression in South Asian mothers in Great Britain has shown they experienced ‘culture clashes’, leading to feelings of being misunderstood by healthcare professionals, and thus notable difficulties in reporting mental health symptoms during the perinatal period [23]. Other studies have found migrant and ethnic minority women, in general, have a higher risk of anxiety symptoms [7], and may experience barriers related to social deprivation according to the Index of Multiple Deprivation (IMD; i.e., Income, Employment; Education; Skills and Training; Health and Disability; Crime; Barriers to Housing Services and Living Environment) [25]. Research analysing Black Caribbean women’s perceptions of perinatal mental health care found they were less likely to engage with mental health (and general health) services in the perinatal period if they experienced poor physical healthcare or care deemed lacking in compassion [14]. In the US, use of psychotropic medication has been suggested as another barrier for minority ethnic women, as their preferences with regards to mental health care interventions were found to prioritize faith and social support over the use of medication [26].

The need for culturally sensitive mental health services and acknowledgement of cultural concepts of distress have been widely recognized as a prerequisite for enabling access and engagement of women from minority ethnic groups [27, 28]. To fully understand the barriers these women experience to access and engage with perinatal mental health care, services need to focus more on women’s wider cultural, social and family context [29].

The need for improved access to perinatal mental health services has been widely recognised in the UK in recent years. The National Health Service (NHS) in England has attempted to address issues with access and limited provision for perinatal mental health support by creating a mental health implementation plan [30], in-line with the NHS Long-Term Plan [31]. It includes proposals for increased funding for community-based mental health care, specialist inpatient care for mothers and babies, as well as the recent increase of service provision to two years postnatal, instead of the previous one year. These steps aim to improve access to perinatal mental health services across the UK, potentially reducing the risk of mother and infant morbidity and mortality, and ensuring continuity of care throughout the perinatal period; alongside other guidance from the National Institute for Health and Care Excellence (NICE) [32]. This study aims to contribute to this goal by exploring the multi-level barriers Black, Asian, and minority ethnic women experience when accessing mental health services in the perinatal period.

## Methods

### Setting

This study was conducted within a community perinatal mental health service in South London, covering an area of London where an estimated 17–34% of the population are from minority ethnic backgrounds [33–35]. The service covers three boroughs in South London: The London Boroughs of Bromley and Bexley, and The Royal Borough of Greenwich. The number of minority ethnic individuals living in these boroughs are expected to increase [33–35], and across the three boroughs, 27% (Bexley), 26% (Bromley), and 61% (Greenwich) of the local population are classified as being deprived, based on the Index of Multiple Deprivation (IMD) [36]. The service offers specialist mental health assessment, care, and treatment for women who are planning a pregnancy, are currently pregnant, or are up to twelve months postpartum. Referrals are accepted from a range of healthcare professionals, including midwives, obstetricians, health visitors, general practitioners (’family doctors’), primary mental health services (commonly referred to as Improving Access to Psychological Therapies [IAPT] services) and other secondary mental health services. The service does not offer crisis or emergency care.

### Study Design

A qualitative research design, using Thematic Analysis, was employed to evaluate minority ethnic women’s experiences of access to and engagement with a community perinatal mental health service in South London. Ethical approval was granted by the Research and Development Office of Oxleas NHS Foundation Trust (approval date: 11/07/2019, Datix reg: 1056). An interview method was utilised in accordance with relevant guidelines and regulations, with the interview schedule [Additional File 1] having been approved by the Research and Development Office. Informed consent was obtained from all study participants, electronically, in writing before the date of the interview, and participants were made aware of their right to withdraw.

Women who self-identified as being from a minority ethnic group were purposefully selected. Topics covered during the interviews included women’s experiences of accessing and engaging with perinatal mental health care and by extension a range of mental health, community, and maternity services within the NHS. In this study, we use the terms ‘minority ethnic’ or ‘ethnic minority’ (where appropriate) to refer to all the women who participated. When we present data from individual women, we provide their ethnicity, as they self-defined it. We recognise ethnic and cultural backgrounds are more nuanced than the broad groupings often provided, however for the purposes of this study, we utilise this terminology as it is widely used and accepted by the academic and public policy communities in the UK. We acknowledge, however, that this terminology may not resonate with everyone, but hope it demonstrates sensitivity around this complex issue of racial, ethnic, and cultural identities.

### Participants and Recruitment

Recruitment took place between May and September 2020. Participants (*N* = 18) who met the inclusion criteria, were identified and discussed during regular clinical multi-disciplinary team meetings. Inclusion criteria meant women had to 1) self-identify as being from a minority ethnic group (Black, Asian, or any other minority ethnic backgrounds, including White Other); 2) be under the care of the community perinatal mental health service (i.e. pregnant up to one year after the birth of their baby); 3) were over 18yrs of age at the time of recruitment. At a first stage, they were informed about the aims of the study by their designated healthcare professional within the community perinatal mental health service. If they were interested in participating, a member of the study team based within the service (SP), would contact them to explain and discuss further details of the study. Consent was taken via a written consent form prior to interview. It was made clear that women’s decision to participate or not to participate would not affect their clinical care in any way. Of those service users that were approached, four women declined to participate in the study.

### Data Collection

An interview schedule (see Additional File 1) was developed (AE, SP) to explore women’s experiences of perinatal mental health services and included topics of access to and engagement with a community perinatal mental health service. The interview schedule was subjected to internal sense-checking, through consultation and supervision with senior staff from the service (SR, SS) and with an academic psychologist with expertise in qualitative methods (SAS). One researcher – who was a trained psychology assistant with experience in working with ethnic minority populations, conducted all the interviews (SP), with consultation and supervision provided throughout the interview process by more senior members of the team (AE, SAS, SR, LMH) [37–39]. Interviews were conducted via telephone, and were semi-structured, which allowed for the same topics to be asked of all participants, but with the flexibility in questioning style to follow-up on points made by individuals [40]. Participants were recruited until thematic saturation was established [41], whereby no new themes were being identified through the analysis when new data (interview transcripts) were added. Participants provided written (electronic) informed consent prior to the interviews commencing, and although most demographics were gathered from their clinical notes, ethnicity was recorded as per participants’ self-identification, in response to the question: “Could you tell me the ethnicity with which you identify?”. Interviews lasted between 25–80 min (M_Time_ = 46 min) and were audio-recorded, after which they were transcribed verbatim, and anonymised transcripts were stored in a secure folder, only accessible by members of the research team. Participants were reimbursed for their time through vouchers to the value of £25.

### Data Analysis

Interview data were analysed in NVivo [42] using a thematic analysis, which follows methodical process of dataset familiarisation, initial coding, searching for themes, reviewing themes, and finally defining and naming themes [43]. The present analysis reports on an empirical exploration of the impact of ethnicity and cultural differences on accessing and engaging with perinatal mental health care. Data analysis was conducted by one researcher – a non-clinical psychologist (SP), with a confirmatory coding of ~ 20% of transcripts undertaken by another researcher – a clinical perinatal mental health midwife (KDB), ensuring conceptual inter-rater reliability across codes and themes [44]. The cohesion, completeness, and meaningfulness of codes and final themes were checked, discussed, and agreed through regular consultation with the wider study team (AE, SAS, KDB, SS, SR, LMH), throughout data collection and analysis stages. Rigour was maintained in thematic analysis and in our study by reflexively engaging with data and analytical processes throughout the different steps of analysis as detailed above. This ensured any biases brought to the data by analysts were identified and removed iteratively during the inductive analysis.

## Results

Half of the women participating in the study self-identified as Black or Black British (*n* = 8), 4 women as Asian or Asian British, 2 as Arab and 4 as Mixed Other or White Other. Women ranged in age between 19 and 46 years at the time of the interview, with a mean age of 33.4 years. 17 participants were interviewed during the postnatal period, and one during pregnancy. One third of participants were first-time mothers (*n* = 6). Women’s primary diagnosis -as recorded in their mental health care records – included Bipolar Affective Disorder (*n* = 2), Anxiety (*n* = 3), Trauma-Related Diagnoses (including Post-Traumatic Stress Disorder [PTSD] and Emotionally Unstable Personality Disorder) (*n* = 5), Depression (including post-natal and recurrent) (*n* = 6) and Psychotic Disorders (*n* = 2). Full demographic information can be found in Table 1.

**Table 1:** Participant Demographics

**Characteristics**	*n*
**Ethnicity**	
Black/Black British	8
Asian/Asian British	4
Arab	2
Mixed Other	2
White Other	2
**Age: (Mean** = 33.4)	
** ≤ **25	2
26–30	3
31–35	5
36–41	7
42–46	1
**Number of dependents**	
1 child	6
2 children	3
3 children	7
≥ 4 children	2
**Primary diagnosis**	
Bipolar Affective Disorder	2
Anxiety	3
Trauma-Related Diagnoses (incl. PTSD and EUPD)	5
Depression (including post-natal and recurrent)	6
Schizophrenia type disorders	2
**Perinatal status**	
Pregnant	1
Postnatal	17

Qualitative analysis of the dataset identified three overarching themes and respective further sub-themes, are presented in Table 2, with the most illustrative quotations presented in the text and supplementary quotations in Table 3, at the end of the results section*.* Each quotation is presented with its corresponding participant identifier and their self-identified ethnic background.

1. **Expectations and Experiences of Womanhood as an Ethnic Minority**

**Table 2:** Summary of Themes

Overarching Theme	Sub-Themes
1. Expectations and Experiences of Womanhood as an Ethnic Minority	i.Shame and Guilt in Motherhood ii.Women as Caregivers iii.Perceived to Be Strong and Often Dismissed
2. Family and Community Influences	i.Blind Faith in the Medical Profession ii.Family and Community Beliefs about Mental Health and Care iii.Intergenerational Trauma and Family Dynamics
3.Cultural Understanding, Empowerment, and Validation	i.The Importance of Understanding Cultural Differences ii.The Power of Validation, Reassurance, and Support

**Table 3:** Supplementary Quotations

1. Expectations and Experiences of Womanhood as an Ethnic Minority	2. Family and Community Influences	3. Cultural Understanding, Empowerment, and Validation
I. Shame and Guilt in Motherhood “Because I went back to placement when he [the baby] was nine weeks old, […] I felt so guilty because I felt like I was missing out on him, on his upbringing and I thought that maybe I’d put university or my ambitions first and I didn’t like how it made me feel.” (Participant 12, Black British) “It is hard to talk to people because people make you feel like you’re a bad mum. They think you are complaining, and you don’t want to complain to the wrong person because some people can’t get pregnant, some people can’t have babies. The last thing they want to hear is someone complaining about being pregnant because it is the most beautiful thing in the world, but it is hard.” (Participant 17, Mixed Other) “I thought it was my fault because he [the baby] had meconium […]. It stuck his lungs together and there was a point I thought it was my fault.” (Participant 8, Mixed Other) “I didn’t tell everyone what I was going through, I just kept it to a handful of people because I was ashamed, of saying ‘I don’t like this pregnancy, I don’t like this baby’.” (Participant 9, Arab) II. Women as Caregivers “After that, I tried always to not be so paranoid about how the house is and if the cooking is done, be more relaxed and think of myself and baby, and not about the household or work or anything else. I was just a bit more considerate to myself.” (Participant 14, Black British) “Sometimes I am suffering a lot because of the headaches. […] And I’m crying and my eldest son, he’s asking why is mummy always crying? Grown-ups are not allowed to cry. So well, it’s difficult to cry in from of them. Most of the time I’m going into the bathroom.” (Participant 2, Asian) III. Perceived to Be Strong and Often Dismissed “I think part of the thing with a woman, they don’t like to admit until it’s really bad. (Participant 10, White Other) “I’m the kind of person that keeps things to myself a lot. If something is really wrong, I would say but if it’s smaller, I will keep it to myself.” (Participant 16, White Other)	I. Blind Faith in the Medical Profession “That’s why professional opinions are so important because it is balanced. A professional person will never tell you what to do. They will guide you and that is what I appreciate.” (Participant 17, Mixed Other) “I would recommend it to them [other women in similar positions] and for them to have more support from the Consultant.” (Participant 4, British Asian) II. Family and Community Beliefs about Mental Health and Care “It’s just because obviously I was brought up in a Caribbean Pentecostal household, so it’s very hard for them to talk about mental health.” (Participant 11, Black British) “In terms of the Arabic culture, there’s very little understanding around mental health, even less so around maternal mental health.” (Participant 13, Arab) “You don’t talk about your mental health. Nobody has mental health, like you just don’t talk about it.” (Participant 15, British Asian) “They [my parents] are supportive, but I wouldn’t necessarily talk to them about my psychological problems, because they would tend to just worry and wouldn’t know how to help, and they wouldn’t understand that you can seek this help and all that.” (Participant 5, British Asian) III. Intergenerational Trauma and Family Dynamics “I will be honest with you; I have seen a few things and I believe a lot of my mental health came from my bloody dad. He is as mad as anything.” (Participant 17, Mixed Other) “You are asking about my family, including my parents in Sri Lanka, with my sister and my elder brother. I am not contacting them, but I only contacted my brothers […]. I’m only contacting two of them over the phone, sometime over email and they, I mean, they’re contacting my parents and I’m getting information through them. I am not straight contacting them [my parents].” (Participant 2, Asian)	I. The Importance of Understanding Cultural Differences “For me, a plus was that most of the people that I came in contact with were Black, or some ethnic background, and to me that’s quite important because they would be able to understand my lived experience.” (Participant 12, Black British) “The perinatal nurse was, I think, from Nigeria. We had a similar understanding of things because we are from similar backgrounds culturally. And that was good because we understood each other on many aspects, yes.” (Participant 9, Arab) “As a Black woman, that was really quite nice to see other women of ethnic minorities in a professional field to do with mental health because there can be more stigma in our communities about that stuff. That felt very good. I did feel a bit sad when the Black mental health nurse left. […] I did miss that presence of having another person who was an ethnic minority. I missed their presence in the service.” (Participant 7, Black British) “Understanding African culture is very different to West Indian culture and to Somalian culture, understanding and recognising there might need to be different things in place.” (Participant 15, British Asian) “Maybe that’s the problem with the system because I was married to an African man and there his way of doing things is slightly different” (Participant 3, Black British) II. The Power of Validation, Reassurance, and Support “He’s seen me through all of the stages of my mental health struggle. He was definitely the catalyst for me seeking help originally. We’ve been together going to be, well, nine years, so it’s been a long journey. Yeah. And he’s definitely not going anywhere because I know that it’s been a challenge and I have to take that really seriously […]. I love him and he’s been… he just really took it on the chin and he really stepped up to it.” (Participant 1, Black British) “I didn’t want to go out, I didn’t want to see anyone. […] When I am feeling like that, I usually go to my brother’s house. I stay there for two weeks, or one week or ten days, until I feel better to come home.” (Participant 18, Black British) “When I was pregnant I wasn’t quite sure whether I was gonna keep the baby… I was still quite upset and things like that. It helped me with that. But contacting the perinatal team they reassured me that if I had the baby it wouldn’t be taken away… so yeah that was good” (Participant 6, Black British)

The first overarching theme related to the expectations of women and being a mother, how this was (or was not) influenced by the experiences of being from a minority ethnic background, and in turn the impact this had on accessing mental healthcare.

i. Shame and Guilt in Motherhood

A vast majority of participants described feelings of shame arising from difficulties with their mental health in the perinatal period, not enjoying the experience of motherhood and a sense of a loss of identity and how this prevented them from seeking help.

“I felt hugely, hugely ashamed of how I was feeling, and that stopped me from accessing services. And I don’t think that’s necessarily the Arabic culture influencing that, I think that’s just the culture of motherhood” (Participant 13, Arabic)

Many participants described emotional difficulties when feeling unable to fulfil certain expectations around motherhood, such as breastfeeding and how these exacerbated feelings of shame and guilt as a mother:

“I used to feel bad I stopped breastfeeding when he was 4 months, I think I felt bad that I did. And then I thought that I was not a good mum” (Participant 14, Black British)

ii. Women as Caregivers

Several participants described the impact of their emotional responsibilities as a caregiver, their role within their family unit and extended family, and the shift in roles from daughter and sister to wife and mother.

“…because I’d just got married, there was a shift from living at home with my sisters and my mum, and also I’m the eldest so my role changed and…they [my family] felt like I was doing a lot for my husband and I’d forgotten about them.” (Participant 12, Black British)

Participants described being expected to carry on with their domestic responsibilities, in addition to childcare, despite it being contraindicated for them to do so and at a time when participants needed to focus on their own recovery.

“On the 6th day after my c-section I had to carry on with all the housework and the midwife was telling me you shouldn’t be doing this because of risk of hernia etc. but I had no other choice” (Participant 4, British Asian)

iii. Perceived to be Strong and Often Dismissed

Several participants described their past experiences of being dismissed by primary healthcare professionals, such as their GP or midwife, when raising concerns about their emotional well-being during routine appointments. They spoke of a reluctance to then ask for help until their mental health difficulties had reached a breaking point, often minimizing the true severity of how they were feeling. This arose within the cultural context of participants feeling as they must be perceived to be strong and may be considered weak for not being able to cope with the difficulties they may be facing.

“But I was worried about how I was going to be perceived …there’s always a thing about being a strong black woman, and sometimes that’s not always the case. We’re not as strong as society expects us to be” (Participant 12, Black British)

For some participants, ideas of being strong was closely interlinked to cultural ideas about women’s strength and spiritual and religious practice.

“…the whole thing of having a psychiatrist and therapy and all that is not…in that culture that is…you have to be a strong person. You pray, try and pray about it. Maybe you’re not religious enough, maybe you’re not spiritual enough, maybe you have to have a stronger faith” (Participant 9, Arab)

Participants from Asian backgrounds frequently described being socialised to minimise conventional overt expressions of physical and emotional pain or suffering. This was, at times, reinforced in services by staff not recognising the cultural differences in how pain is expressed.

“I was in a lot of pain [after an ectopic pregnancy] but…I had to fight to keep my painkillers and they [doctors and nurses on the gynaecology ward] are like oh you seem fine and I’m just like you can’t send me home without painkillers” (Participant 15, British Asian)

2. Family and Community Influences

Many participants discussed how beliefs stemming from their family and community background and community had influenced their views of mental health, experience of services, and the impact this had on their access to care.

i. Blind Faith in the Medical Profession

Participants identifying as Asian often reported a deep-rooted faith in the ability of medical doctors, to whom a higher level of competence was attributed compared to other professionals. This meant their families were more trusting of medical doctors and less likely to contradict their advice.

“There’s kind of a blind faith in medical professionals and that’s definitely cultural. Definitely in the Indian community” (Participant 15, British Asian)

They also reported they were more likely to feel reassured when given health advice by medical doctors and at times would feel disappointed or dismissed when support was given by other, non-medical, professionals.

“I felt more comfortable in myself that the doctor has seen him [the baby] and reviewed him.” (Participant 12, Black British)

“I think earlier on I was expecting more [the doctor] to make contact with me. I was expecting a psychiatrist talking to me at the beginning, but it didn’t happen…what happened was the different one came in, she was a psychologist” (Participant 9, Arab)

ii. Family and Community Beliefs about Mental Health and Care

Many participants described a lack of recognition and understanding of perinatal mental health in their communities. Mental health symptoms were often dismissed by family, and when this was not the case, there was a fear of accessing and engaging openly with services, which increased the risk of an escalation in mental health symptoms. Participants from all minority ethnic backgrounds discussed how mental health was not spoken about and there was a lack of understanding by family and community members around the symptoms and causes for mental health difficulties.

“His mum… had this African mentality in the background, like ‘Oh, you don’t want to go [to the GP]. Okay, you’re fine, don’t worry it’s nothing’” (Participant 12, Black British)

Participants would find these comments blameful, dismissive and hurtful, which aggravated their existing emotional distress.

“They [my family] would just say… ‘there's nothing wrong with you, you have such a beautiful life, you have a child, you have a nice husband, you have a nice house and you're a beautiful girl, so what is wrong with you?’ I used to hear a lot of comments that were very hurtful.” (Participant 14, Black British)

Participants who identified as Black or Mixed ethnicity spoke of family members discouraging disclosures about mental health for fear of the implications, such as social care involvement and removal of their baby at birth.

“…a lot of women don’t go to their GP to tell them they are pregnant so a lot of it does go under the radar, hence a lot of babies are taken at birth because they haven’t communicated during their pregnancy” (Participant 17, Mixed Other)

Fear was also present with regards to medication as a potential line of treatment for their mental health difficulties.

“I was scared of taking the medication because I just didn’t want to be on something that I’m going to rely on… It took me months to actually start those antidepressants. I was taking one or two and then stopping completely and being scared” (Participant 9, Arab)

iii. Inter-generational Trauma and Family Dynamics

Several participants discussed finding out about family mental health problems through their own experiences as well as describing fractured family dynamics. In some cases, participants described not having any contact with family from a very young age, leading to traumatic upbringings and childhood in care.

“I left my family four years ago and I don’t have any contact with them” (Participant 16, White Other)

Others described challenging family dynamics about raising their child as a single parent, leading to inter-generational conflict around amending this and what parental behaviour is deemed acceptable.

“I felt a pressure from my family to try and make things work with her dad. I felt very judged by my family. The same went for after she was born. It amazed me how little her dad had to do for people to think that he deserved more chances or that he was a good dad” (Participant 7, Black British)

3. Cultural Understanding, Empowerment, and Validation

The final theme related to participants’ experiences of clinicians’ cultural understanding, feelings of empowerment and validation and how these acted as facilitators to accessing and engaging with perinatal mental health care.

i. The Importance of Understanding Cultural Differences

Women described they had been met in previous encounters with the healthcare system with a lack of cultural awareness. They had been discriminated against or judged on the basis of their ethnicity and culture and reported negative experiences in relation to their ethnicity, leading to having negative perceptions of the NHS as a system.

“Generally, with NHS services I notice microaggressions all the time…are not overt, but it will be noticing the difference in the way somebody either greets me or I find a lot of receptionists in hospitals will ignore you” (Participant 7, Black British)

Participants reported a wide range of negative experiences, which had led to treatment delays and missed opportunities for care when they disclosed their mental health difficulties in maternity as well as in mental health services. Participants felt their disclosures were dismissed by healthcare professionals, most often at primary care level.

“I was [in a] really, really in a dark place they [the health visitors] told me to eat vitamins, instead of like listening to what I was saying” (Participant 10, White Other)

Others described how professionals were reluctant to refer them to the appropriate mental health service and how the onus to self-refer was completely on them.

“I think the service is amazing once you’re in it, but doctors [General Practitioners] are very reluctant I found, when you’re pregnant. The doctor I saw initially referred me to [a mental health charity], you have to self-refer which doing yourself is not easy. Then you wait for a call, then an interview, it’s all difficult to do when you’re in a bad place.” (Participant 5, British Asian)

To overcome these previous negative experiences, participants described how they felt it was important for their clinicians to be aware of their specific culture and how they related to them as this made them more likely to engage with that clinician.

“Understanding cultural differences is a big thing. In our society in Bangladesh, we are just with partner, we accept a lot of things which are not accepted in British society so if my counsellor doesn’t understand, they [the counsellor] cannot help me” (Participant 4, British Asian)

Others described how cultural awareness and physical resemblance are essential to feel understood by healthcare staff, especially in the context of a perinatal therapeutic relationship.

“With the perinatal, with culture… if you was a black lady with mental health, maybe you wouldn’t want to talk to a white girl…you think white privilege is there and as a black lady you think: does she understand? Does she get me?” (Participant 17, Black British)

ii. The Power of Validation, Reassurance, and Support

Participants described the difference it made when they had support from family and friends during the perinatal period. They also valued the professional support they had received as it made them feel empowered when they didn’t feel worthy of help.

“I didn’t feel at first, worthy? But I think like after I had been and I was reassured that I hadn’t been accepted into the service if I didn’t need it but it’s been a long process I think I’ve constantly thought maybe I was draining the resources” (Participant 10, White Other)

Some women reflected on their own experience and encouraged women to seek and accept help earlier.

“I think the main advice is to get help quite early on…to remove that shame that comes with it all…that was one of the bigger reasons I didn’t fully disclose what was going on.” (Participant 13, Arab)

The insight that women were not alone, but others were going through a similar experience, was reported as being highly important. Women reported to feel reassured when joining the peer support group, which was available to all participants in our sample and where they were able to meet other women they related to.

“So maybe these things are good to have because with peer support, you feel that you’re not alone. Because it can be quite lonely can’t it.” (Participant 10, White Other)

For some women, this sense of camaraderie became increasingly important and powerful as a means to support each other through good times and bad times.

“Like I think four-to-five women who are regulars and we seem to have really opened up to each other and have developed a supportive group and celebrate each other’s wins and commiserate the down times” (Participant 15, British Asian)

Professional reassurance and validation were equally important, especially when they felt their feelings and concerns had previously been dismissed.

“One thing that was really important to me was that feeling when he [the psychiatrist] kept saying I treat so many people in similar situations…no one talks about it. The reassurance…it makes you feel like this is something that people can go through and you’re not the only one” (Participant 5, British Asian)

## Discussion

Overall, our findings highlight how minority ethnic women struggle with their own perceptions of what it means to become or to be a ‘good mother’, especially when in receipt of a mental health diagnosis [45, 46]. Under our first theme ‘Expectations and Experiences of Womanhood as an Ethnic Minority’, we demonstrated that women’s perceptions are closely interlinked with their cultural beliefs and influences. Women from Black and Asian backgrounds reported feeling they had to engage with the illusion of being strong for fear of how they may otherwise be perceived. This expectation, by implication, then meant any symptoms of mental ill health acted as a proxy for lesser mental strength or ability to cope [22]. For Black women, this could give further evidence for endorsement of the ‘strong black woman’ stereotype potentially making them more susceptible to stress and depressive symptoms [47, 48].

In addition to barriers linked with cultural beliefs about motherhood specific to women of ethnic minority backgrounds, participants also appeared to experience similar barriers to seeking help as previously reported for women in the perinatal period from non-minoritized backgrounds – particularly stigma and fear of accessing services and treatment [11, 14]. Our analysis showed women expressed anxiety about taking medication in the perinatal period and were more likely to prefer holistic approaches and talking therapies. Although there is a lack of research on this in the perinatal period, it supports the existing evidence that those from minority ethnic backgrounds are less likely to endorse medication in their general mental healthcare preferences [49]. This is pertinent as our research also highlighted difficulties in medication adherence which can potentially exacerbate mental ill-health and may lead to higher rates of involuntary admission to psychiatric hospitals in the perinatal period amongst minority ethnic women [50]. Providing tailored advice and preconception support [51, 52] and addressing medication concerns may be one way to improve these outcomes [50].

As demonstrated under our second theme (Family and Community Influences), strong family and community beliefs surrounding mental health and (mental) healthcare could further compound barriers to accessing mental health support for minority ethnic women who are struggling with their mental health in the perinatal period. This challenges the common view that women seek informal emotional support during the perinatal period from family and friends as a first resource, before they call upon more formal, professional, mental health support [53]. In our sample, many participants felt unable to disclose their mental health difficulties to their families or would minimise the extent of their struggles, out of fear of being dismissed and not being a *‘good enough’* mother or woman [46]. However, these findings do not negate the widely accepted importance of family and other social support as a protective factor in the perinatal period [54], as is evident from the findings under our third and final theme (Cultural Understanding, Empowerment, and Validation). The importance of social support during the recovery from perinatal mental health difficulties [49, 55, 56] was endorsed across all minority ethnic backgrounds within our sample. Previous research has associated family involvement in Black women’s pregnancies with lower depressive symptomatology and may act as a protective factor [57]. Participants in our study also highlighted their mental health would have been far worse without the invaluable practical and emotional support from their partner and/or father of their baby. Another valuable resource of social support highlighted in our findings is that of other women going through a similar experience. Peer support has been found to be hugely beneficial in a perinatal context, not just for women who face mental health difficulties [58, 59] but also for women with additional social vulnerabilities during this time [60].

Furthermore, the findings emphasise the importance of representation of healthcare professionals with whom participants could identify, i.e. healthcare professionals from minority ethnic backgrounds. In absence thereof, our findings highlight the importance of healthcare professionals who show cultural awareness and humility to overcome this lack of representation in the workforce. This reinforces findings from the existing research regarding racial and ethnic disparities in accessing healthcare and treatment [11–15]. Women highlighted the need to provide healthcare staff with cultural awareness so as they can support women to facilitate access to services as well as the overall experience within them. The concept of ‘cultural humility’, introduced by Tervalon and Murray-Garcia in 1998, is critical in this context, as it requires a commitment from healthcare professionals to self-evaluation and self-critique, and to acknowledge there are other models of distress alongside Western models of psychology and psychiatry [28, 61]. To embed cultural humility in mental health services, critical re-thinking of healthcare training and service delivery is needed. Evidence suggests staff in secondary mental health care and midwifery lack training in the needs of minority ethnic women, particularly the healthcare needs of Black women [62]. Robinson et al. (2021) recommends embedding cultural humility in the healthcare workforce using the 5Rs of Cultural Humility (Reflection, Respect, Regard, Relevance and Resilience) to address implicit bias against minority populations and to facilitate organisational culture shifts toward cultural humility in healthcare [63]. In addition, primary care services delivering psychological interventions have highlighted the need for tailoring interventions to minority ethnic populations and providing staff with training to address the current gaps in knowledge of culture and cultural concepts of distress, as well as tackling structural inequalities at service level [19]. To achieve this, co-production has been widely accepted as a successful strategy to reduce mental health inequalities for people from minority ethnic backgrounds [64] and has been adopted by both government, professional bodies and patient groups as the way forward to improve access and engagement in mental healthcare [65].

### Strengths, Limitations, and Future Directions

This study provides an in-depth, qualitative understanding of a group of minority ethnic women in South London. The participants interviewed in this study self-identified as ‘being from a minority ethnic background’. This included both women who were born in the UK as well as those who migrated to the UK at a later age. Therefore, our findings do not distinguish between women born in the UK or with migrant status. None of the women had refugee status, and given the specific challenges women with refugee status face, they are not within scope of this study [7]. As such the findings may not be more widely generalisable or representative of the experiences of women in other settings or women who are not ethnic minorities in those other settings. Participants interviewed had high levels of social complexity (such as housing difficulties, lower socio-economic backgrounds, and/or victim of trafficking) which, in itself, can provide additional barriers to service engagement and indeed, participation in research. Further, due to the COVID-19 pandemic and the Government-mandated ‘lockdown’ which was imposed in the UK during the time of recruiting for participants, interviews were conducted remotely via telephone. This meant it was more challenging for some participants to engage with the research, and it may have been more challenging to establish a rapport between researcher and participant due to telephone interview set-up, limited access to internet connections to arrange the interview and credit for mobile phones, which may have affected the depth of discussion. These types of data poverty are essential issues for research teams to address before commencing future research [66]. A strength of the study is the use of qualitative methodology with semi-structured interviews, allowing women to speak freely without being restricted to pre-assigned ideas about their beliefs and feelings about the topics discussed [67]. Given the limited research in this area, future research should be aimed at supporting services to conduct holistic assessments exploring the family network and support, whilst obtaining true insight into women’s individual contexts.

## Conclusion

Our study provides new insight into the experiences of minority ethnic women when accessing and engaging with mental health services in a perinatal context. Women identified barriers to accessing support during this time on an individual, familial, social network, and societal level. The tension between cultural beliefs and expectations of woman – and motherhood – on one hand and their day-to-day lived experience of mental health difficulties, on the other, compounded their existing psychological distress and undermined their self-worth as a mother.

Furthermore, the understanding of perinatal mental health pathways has been cited as a key way of helping women receive specialist advice, reach the appropriate services at the right time, and respond effectively to any difficulties they may be experiencing[59]. These needs should be carefully considered for minority ethnic women who may present with mental health difficulties in different ways. This cannot be achieved without increased training for healthcare professionals in cultural humility and by adoption of co-production in mental healthcare, in order to address systemic health inequalities and to tailor and improve mental health care to fully meet the needs of the communities they serve.

## Patient and Public Involvement and Engagement

This programme of work has been presented to, and received input from the National Institute for Health Research Applied Research Collaboration [NIHR ARC] South London Patient and Public Involvement and Engagement [PPIE] meeting for Maternity and Perinatal Mental Health Research (July 2020; June 2021), which has a focus on co-morbidities, inequalities, and maternal ethnicity. Throughout the course of planning and undertaking this work we have received feedback from both lay and expert stakeholders, including members of the public, those with lived experience, health and social care professionals, researchers, and policy makers.

## Supplementary Information

**Additional file 1.** Interview Schedule

## Data Availability

The datasets generated and/or analysed during this study are not publicly available due to the sensitive nature of the interviews. Summary data may be shared from the corresponding author upon reasonable request, when compliant with ethical regulation of this study.

## Abbreviations

ACE: Adverse Childhood Experiences
ARC: Applied Research Collaboration
EUPD: Emotionally Unstable Personality Disorder
GP: General Practitioner
IAPT: Improving Access to Psychological Therapies
NHS: National Health Service
NICE: National Institute for Health and Care Excellence
NIHR: National Institute for Health and Care Research
PPIE: Patient and Public Involvement and Engagement
PTSD: Post-Traumatic Stress Disorder
UK: United Kingdom
US: United States (of America)
