# Supplementary material for: A qualitative study of minority ethnic women’s experiences of access to and engagement with perinatal mental health care
Source: BMC Pregnancy Childbirth. 2022 May 18;22:421. doi: 10.1186/s12884-022-04698-9 (PMC9116695; doi:10.1186/s12884-022-04698-9)
Supplement: Supplementary file 1 — Additional file 1. Interview Schedule [file 12884_2022_4698_MOESM1_ESM.docx]

**Additional File 1 – Interview Schedule**

Firstly, I would like to thank you for taking part in this service evaluation. As this is a new service and we are always looking at ways we can improve, we would like to find out about your experiences in accessing XX Perinatal Mental Health services. We would like to find out about the experiences you had during or after your pregnancy. We are interested in individual coping skills and the care individuals have received. It doesn’t matter if you have or haven’t accessed services, we are just interested in your experiences.

We are keen to hear your views and there are no right or wrong answers. Your answers will be anonymised and will not affect the care you are receiving from the services. The interview typically lasts around 45 minutes.

Demographics questions - Could you tell me the ethnicity with which you identify?

*Introduction & well-being*

What does wellbeing mean to you?

How would you describe your well-being during pregnancy/ being a new mother?

*Contact with XX Perinatal Mental Health services*

Are you currently in contact with services or have you been discharged? Is discharge upcoming – how do you feel about that?

When did you first come into contact with XX Perinatal Mental Health Service? (Prompt was it too early/ or late– if referral was late in pregnancy/antenatally why was that?)

Who did you see and what has been your experience of initial contact?

What did you expect from the service? Did it meet your expectations?

What kind of support were you offered?

Did you feel the service met the needs of your individual/ personal situation?

Do you have any suggestions for what you think might work for you? & Is there anything you would have liked to be offered?

Culture & diversity

What does culture mean to you?

Has your culture/ race/ family history or background / where you grew up affected the way you experience services?

Did you feel the service met the needs of your cultural situation? e.g. your language or way of life?

Can you describe in what ways the service met/did not meet these needs?

Do you think your culture or ethnicity has ever impacted on the support you have received? (in this service and others – if able to elaborate)

When working with a healthcare professional, would you prefer to speak to someone of your own ethnicity/ who shares the same culture as you?

What qualities in healthcare professionals are important?

*Mental health*

Could you tell us about any struggles you may have had during/after pregnancy? (Prompt, e.g. mental health)

Do you have any self-help strategies that you use to help manage these difficulties?

Which strategies have you found helpful/less helpful?

How has your family/partner responded to your pregnancy and any difficulties you may have faced?

*Barriers to access*

Have you had any experiences of housing/migration that have affected how you are able to access support and/ or services?

Where or who would you go to if you were experiencing worries or emotional difficulties? – (What would be your first contact)

Have you experienced any difficulties attending appointments? (prompt: physical health, transport, financial, work, childcare, etc.)

What do you think might make it easier for people to attend appointments?

*COVID- 19 Impact*

How has coronavirus impacted your ability to contact or engage in services? (our service but also others – midwife appointments etc.)

Did you attend 1:1 therapy or groups before- have these been affected?

Have you had any telephone assessments/ scheduled appointments over the phone? If YES – how have you found them compared to face-to-face contact?

IF YES, were you offered an alternative? Would you have liked to have been offered remote telephone/ video appointments?

Do you have any suggestions for what would help you access support remotely?

*Facilitators to access*

What advice would you have for us to improve the service or access to the service?

What care/services would you like to see us provide?

What advice would you have for women in a similar situation to you?

**Interview close**

I’ve now come to the end of my questions. Thank you for sharing your experiences with us.

Do you have anything else you’d like to add that I haven’t asked you about?

Do you have any questions for me?

Ask if they’d like to be contacted for future research
